# Symptom Management in Patients with Stage 5 CKD Opting for Conservative Management

**DOI:** 10.3390/healthcare4040072

**Published:** 2016-09-22

**Authors:** Sheila Johnston

**Affiliations:** Royal Free Hampstead NHS Foundation Trust, London NW3 2QG, UK; sheilajohnston@nhs.net; Tel.: +044-020-7794-0500 (ext. 36898)

**Keywords:** stage 5 chronic kidney disease, conservative management, supportive care, palliative care, symptom management

## Abstract

Chronic kidney disease (CKD) stages 3–5 now affects 8.5% of adults in the United Kingdom; with 4% of patients expected to reach stage 5 CKD. Increasing numbers of older patients are contributing to the growth of demand of kidney services. With the exception of transplantation, dialysis has been the main form of renal replacement therapy (RRT) for advanced CKD. This elderly population is usually too frail and has many other co-existing medical complaints or co morbidities to undergo transplantation. Dialysis is an invasive treatment, and some frail elderly patients can experience many dialysis related symptoms. An alternative option for these patients is to choose conservative management (CM) of their stage 5 CKD. These patients often have complex supportive and palliative care needs. The frequency, severity and distress caused by symptoms related to stage 5 CKD are often under recognized and under treated. There is a need for early identification and management of symptoms as they present in patients with stage 5 CKD being managed conservatively. Symptom assessment should be focused on anticipating, identifying and alleviating any symptoms. This needs to be incorporated into the regular practice of those managing CM patients.

## 1. Introduction

Stage 3–5 chronic kidney disease (CKD) is common and now affects 8.5% of the UK adult population [1]. Stage 3–5 with disproportionate numbers of older patients contributing to this increase [2,3]. Diabetic kidney disease is the single most common cause of renal failure and accounts for 24% of patients with chronic kidney disease (CKD) in the UK [4]. The National Institute of clinical excellence (NICE) recommend that people with CKD stage 4 and 5 are referred for specialist assessment by a nephrologist. Stages 1–3 can usually be managed by General Practitioners (GP’s) [5]. CKD is often associated with other medical conditions, such as heart disease and diabetes. There is an increased risk of mortality in patients who have advanced CKD.

The lack of specific symptoms can result in people with CKD not being diagnosed or diagnosed when they have advanced stages of CKD. Approximately one third of patients who have the advanced stages of kidney disease have a late referral to kidney services which is associated with an increase in mortality and morbidity [5]. Strategies aimed at early identification of CKD and where possible, preventing the progression to advanced kidney disease are therefore clearly needed [5].

Although renal replacement therapy (RRT) has transformed prognosis of kidney disease, quality of life remains impaired by the disease and there is a significant treatment burden associated with dialysis [6].

Invasive surgical procedures related to haemodialysis access are often required, and each haemodialysis session lasts 4 hours three times per week for the rest of the patient’s life unless a kidney transplant is received.

Peritoneal dialysis (PD) could be seen as a valid treatment option in frail elderly patients as they do not need to travel to haemodialysis units three times per week. Some centers offer an assisted PD service for those patients who have dexterity or cognitive problems impeding their ability to carry out the dialysis. It has however, been suggested that this can have a negative impact on the patient’s family and/or care givers. There is also an increased risk of peritonitis in addition to social isolation as this is a home based therapy [7].

Some recent studies demonstrate the survival on dialysis of this group may be very poor. Treatment has a negative impact on quality of life. Evidence remains sparse [8], however, recently there is growing evidence that dialysis may not prolong life in the elderly with poor functional status and multiple co-morbidities [9,10,11].

Over 2% of the total National Health Service (NHS) budget is spent on RRT [5].The impact of the global recession and the massive public debt in the UK cannot be ignored in the provision of health care today. The NHS continues to face the challenges of austerity and funding cuts. The view is that the NHS will be under continued pressure to provide service improvements with no extra funding over the next five years. Even with continuing improvements in CKD screening services, earlier referral of patients with advanced CKD and where appropriate, provision of supportive care in place of RRT for patients with stage 5 CKD with significant co morbidity, it is believed that the prevalence of RRT will continue to increase [12].

## 2. Conservative Management

An alternative choice for these patients is the option of Conservative Management (CM) in stage 5 CKD. CM refers to management without dialysis which includes active management of the kidney disease to slow further deterioration of kidney function and to minimize complications of the kidney disease [9].

As symptoms escalate and the end of life is closer, some symptoms can be difficult to manage such as fluid overload and lethargy. Patients with stage 5 CKD managed conservatively have significant symptom control needs, similar to those of patients with advanced cancer [8].Kidney service providers have developed innovative services by integrating specialist palliative care and nephrology care, and promoting collaborative working across the two specialties. A central aim of these services is the appropriate management of symptoms as the patient managed without dialysis ultimately deteriorates over time.

Some studies have been carried out to attempt to measure the prevalence and severity of symptoms experienced in CM patients. One study completed a cross-sectional survey of these patients to identify the prevalence and severity of symptoms experienced by CM patients [8]. This study used the Memorial Symptom Assessment Scale Short Form (MSAS-SF) with additional kidney specific symptoms added to the tool. Sixty six patients were recruited and symptoms were reported in more than 33% of the patients. The mean number of symptoms present being 11.58 per patient and included lack of energy, fatigue, pruritus, dyspnea, pain, dry mouth and muscle cramps. Other symptoms identified were sleep disturbances, poor concentration, constipation and nausea. Patients with stage 5 CKD managed conservatively have multiple and significant symptom control needs.

CM patients experience many associated symptoms which can have a negative impact on their well-being and may also carry a heavy symptom burden from other medical conditions such as heart failure or diabetes. Early intervention with medications aimed at treating these symptoms is advisable.

Staff caring for patients with CKD should have to the necessary skills and knowledge to identify symptoms early. They will then need to implement an agreed plan with the patient. This should include where applicable: Prescribing any recommended medications with the aim to arrest or slow any further rate of decline in kidney function and effectively manage the symptoms associated with stage 5 CKD.

As kidney function declines, sodium retention and extra-cellular volume expansion lead to hypertension, and peripheral and pulmonary oedema. Extra circulating fluid increases the amount of fluid in the blood vessels and increases the BP. Diuretics in stage 5 CKD are used to reduce fluid accumulation in both the intracellular and extracellular spaces. Peripheral and pulmonary oedema becomes more problematic when the eGFR falls to less than 10–15 mls/min.

It can be difficult to estimate when a patient is approaching the end of life. A thorough assessment of the patient’s condition and prescribing of medications should be carried out by a senior clinician. All patients approaching the end of life should be prescribed ‘as required’ medications in case they develop symptoms commonly experienced at the end of life; This is defined as anticipatory prescribing. Common symptoms include; pain, terminal restlessness/agitation; and the effect of secretions in the airway.

The Liverpool care pathway (LCP) has now been phased out following an independent government review in 2013 [12]. This was then followed by the publication of the five priorities of care report in 2014 [13]. This report identifies that caring for people who are approaching the end of their life demands compassion, kindness and a skilled application of knowledge. This publication also set out duties and responsibilities of healthcare providers and all NHS Trusts were required to generate individualised care plans to include these priorities. Table 1 [13] below outlines these priorities of care to include anticipatory prescribing recommendations in Table 2 [14] to improve symptoms management.

In the absence of the LCP each practitioner should refer to local protocols and liaise with palliative care teams for any specialist advice [12].

The palliative care team at an acute London Trust has developed the local symptom control guidance for dying patients. This protocol [14] includes anticipatory ‘as required’ prescribing to manage pain; agitation; respiratory secretions; nausea/vomiting; breathlessness.

## 3. Pain

- For those already on background oral opioids but unable to swallow, convert to a continuous subcutaneous syringe driver infusion (the use of which must be thoroughly discussed with patients and their families) using Table 3 below:

**Table 3:** Conversion chart for commonly used opioids (* denotes the trade name of drug).

Codeine 24 h Oral Dose (mg)	Tramadol 24 h Oral/IV Dose (mg)	BuTrans* Patch (mcg/h) Weekly	Transtec * Patch (mcg/h) Twice Weekly	Fentanyl * Patch (mcg/h) Every 3 Days	Oxycodone 24 h Oral Dose (mg)	Oxycodone 24 h Subcutaneous Dose (mg)	Alfentanil 24 h Subcutaneous Dose (mg)
60							
120	50–100	10			7.5	5	0.5
240	150	20		12	15	7.5	1
	200		35	12	20	10	1.5
	200–400		35	25	30	15	2
			52.5	25	45	20	3
			70	37	60	30	4
				37	75	35	5
				50	90	45	6
				62	105	50	7
				62	120	60	8
				75	150	75	10
				100	180	90	12
				125	225	110	15
				150	270	135	18
				175	315	155	21
				200	360	180	24
				250	450	225	30
				500	900	450	60

### 3.1. Analgesia

- If the patient is opioid naïve and unable to take regular oral medication:
  ○ Commence 5–10 mg/24 h morphine sulphate SC via syringe driver.
  ○ Anticipatory prescribing for opioid naive patients: Morphine 2.5–5 mg s/c prn.
  ○ If known to have renal impairment/renal failure/morphine not tolerated, use: Oxycodone 1.25–2.5 mg s/c prn.
- If the patient was already on an oral opioid, convert to the subcutaneous equivalent. (See Palliative Care Opioid conversion guidance in Table 3 [14].
- Most patients require at least low dose opioid analgesia for discomfort in the terminal phase. However if the patient appears comfortable and has not previously required analgesia, prescribe appropriate opioid prn and review regularly. Only start a syringe driver if they are uncomfortable or have required two or more SC doses in the previous 24 h.
- If the patient is taking analgesia via a patch (Fentanyl, Buprenorphine) leave the patch on and add a syringe driver with any extra analgesia required. Contact the Palliative Medicine Team for advice.

### 3.2. Agitation

- Terminal restlessness and agitation is a common symptom at the end of life, studies. Symptoms may include inability to relax, picking at clothing or sheets, confusion and agitation, and trying to climb out of bed.
- Consider potentially reversible causes of agitation:
  ○ Pain
  ○ Urinary retention
  ○ Full rectum
  ○ Nausea
  ○ Temperature
  ○ Cerebral irritability
  ○ Side effects of medication (especially steroids)
- Medication
- 1st Line, Midazolam 10–30 mg SC/24 h via syringe driver PLUS 2.5 mg–5 mg SC prn.
- 2nd Line, Levomepromazine 12.5–50 mg via syringe driver PLUS 1.5 mg prn and contact the palliative care team.

### 3.3. Respiratory Secretions

Management: Table 4 below recommends medications to manage the respiratory secretions in those patients approaching the end of life:

- The noise is often distressing for the patient’s family—reassure them that the secretions are not distressing the patient.
- Reposition patient and stop parenteral fluids—if unsuccessful consider drug treatment.

### 3.4. Nausea/Vomiting

- A PRN anti-emetic should always be prescribed as above for a dying patient even if not previously required as symptoms can vary.
  ○ Haloperidol 1.5 mg SC/po max 10 mg/24 h (antiemetic and anxiolytic).
  ○ If patient has Parkinson’s, use levomepromazine 6.25 mg SC prn instead of haloperidol.
  ○ Cyclizine 50 mg s/c tds prn max 150 mg/24 h (if likely cause drug induced/bowel obstruction/metabolic or intracranial cause).
  ○ OR Metoclopramide 10 mg s/c tds prn max 60 mg/24 h (if likely cause gastric outflow obstruction/gastric stasis).
  ○ If above ineffective, prescribe Levomepromazine 6.25 mg s/c tds prn.
    Caution—cyclizine may precipitate in a syringe driver when combined with hyoscine butylbromide, if this occurs, switch to alternative antiemetic.
- If nausea/vomiting continue please contact the Palliative Care Team.

### 3.5. Dyspnoea

- Opioids or Midazolam can be effective for dyspnoea.
- Start a syringe driver with morphine sulphate 10 mg SC/24 h.
- If distressed, consider Midazolam 10 mg SC/24 h.

### 3.6. Anticonvulsants

- A patient who was on a regular anticonvulsant and who is no longer able to swallow MUST be started on a syringe driver with Midazolam 20 mg SC/24 h. This can be titrated up to 60 mg.
- If a dying patient continues to fit despite Midazolam 60 mg add Phenobarbital 600 and titrate up to 1600 mg via a syringe driver. Give a loading dose of 200–1000 mg IV (give at a rate of 50 mg/min until fitting stops and call the palliative care team.

### 3.7. Nutrition and Hydration

- Oral hydration and nutrition should continue to be offered as tolerated.
- Risk feeding, where there is a risk of aspiration, is appropriate if the patient wishes/appears to enjoy it.
- Parenteral support may be considered where clinically appropriate.
- Always discuss plans where possible with the patient, and family, and clearly documented.

Further advice on symptoms management can be also accessed from www.Kidneysupportive.care.org [15].

The National Service Framework for Renal Services [16] highlights the need for appropriate end-of-life care for kidney patients and the recommends collaborative working between the kidney multi-professional teams, primary care and other services The End-of-life care Strategy [17] has stressed the importance of education, training and continuing professional development for staff.

An example of a successful collaborative working model is the development a joint CM/Palliative Care nurse led clinic. This model has been recognized as being extremely helpful in bridging any knowledge deficit between kidney and palliative care specialist nurses and allowing an integrated approach to delivering palliative and supportive care. The study’s limitations however, are that only small numbers of patients were surveyed and the feedback was informally received. [18,19].

This has allowed CM patients with complex health and social care needs to be reviewed by a CKD clinical nurse specialist (CNS) and a palliative care CNS. This has allowed improved access to specialist palliative care advice regarding symptom management. It has also been an excellent learning opportunity for the CKD CNS’ to improve their skills and knowledge of prescribing symptom relief medications for this group of patients. It is of great importance that healthcare teams caring for patients approaching end of life have the expertise to support this group and education opportunities should be commissioned by providers as a matter of priority.

## 4. Conclusions

Many kidney units are experiencing an increase in the number of frail elderly patient to their service. This can present both challenges and opportunities for renal specialist nurses. These patients may opt to follow a supportive and palliative care pathway and can carry a symptom burden similar to that of cancer patients. It is extremely important that chronic kidney disease specialist nurses can access education and continuing professional development and develop skills in identifying and treating these symptoms. Collaborative working with palliative care teams can provide an excellent learning opportunity for chronic kidney disease specialist nurses in developing the necessary competence and confidence in managing this often complex group of patients. Services should be further developed to ensure we can be responsive to this growing cohort of patients. Care planning should involve healthcare providers across both primary, secondary and community teams to ensure the patient’s management plan is contemporaneous and clearly documented.

## Figures and Tables

**Table 1:** Priorities of care for the dying person.

Priority 1: Recognise: Clinical teams should recognise the possibility that patient may die in the coming days or hours and this is clearly communicated. This communication should include any decisions made and actions taken in accordance with the person’s needs and wishes. These should be reviewed regularly and updated accordingly.
Priority 2 : Communicate: Ensure that sensitive communication takes place between staff and the patient and those people that are important to them, such as family, spouse, friends
Priority 3: Involve: Ensure that the patient, and those more important to them, such as family, spouse, friends, are involved in all decisions about treatment and care to the extent that they want to be
Priority 4: Support: Ensure that the needs of the patients’ families and others are identified as important to the patient are actively sought, respected and met as far as possible
Priority 5: Plan and Do: Agree, co-ordinate and deliver an individualised care plan with the patient, which includes food and drink, symptoms control and psychological, social and spiritual support

**Table 2:** Anticipatory prescribing recommendations [14].

**Prescribe prn:**
1. **Analgesia** (see below) 2. **Anxiolytic/muscle relaxant** Midazolam 2.5–5 mg SC prn maximum hourly 3. **Antiemetic—depending on likely cause of nausea and /or vomiting** Haloperidol 1.5 mg SC/po max 10 mg/24 h (antiemetic and anxiolytic) Cyclizine 50 mg s/c tds prn max 150 mg/24 h (if likely cause drug induced/bowel obstruction/metabolic or intracranial cause) OR Metoclopramide 10 mg s/c tds prn max 60 mg/24 h (if likely cause gastric outflow obstruction/gastric stasis) If above ineffective, prescribe Levomepromazine 6.25 mg s/c tds prn If patient has Parkinson’s, use levomepromazine 6.25 mg SC prn instead of haloperidol 4. **Respiratory secretions** Hyoscine Butylbromide (Buscopan) 20 mg prn maximum 180 mg/24 h Alternative antimuscarinics if above ineffective—Hyoscine hydrobromide 0.4 mg s/c qds prn OR Glycopyrronium 0.2–0.4 mg s/c qds prn
Caution—Cyclizine may precipitate in a syringe driver when combined with other drugs, if this occurs, switch to alternative antiemetic
These should be prescribed for all patients except in cases of known allergy to the above

**Table 4:** Medications to manage respiratory secretions.

**1st line**	Hyoscine Butylbromide (Buscopan) 20 mg prn If secretions do not resolve with repositioning and PRN Buscopan, start Buscopan 60 mg SC/24 h in a syringe driver. The dose can be titrated up to max 180 mg in 24 h as needed.
**2nd line**	If above ineffective, consider Hyoscine hydrobromide 0.4 mg s/c qds prn (this has some sedative effect). This can be used in a syringe driver dose 1.2–2.4 mg/24 h OR Glycopyrronium 0.2–0.4 mg s/c qds prn This can be used in a syringe driver dose 1.2–2.4 mg/24 h

## References

[1] Stevens P.E., Donoghue D.J., de Lusignan S., Van Vlymen J., Klebe B., Middleton R., Hague N., New J., Farmer C.K.T. (2007). Chronic kidney disease management in the United Kingdom: NEOERICA project results. Kidney Int..

[2] Roderick P., Davies R., Jones C., Feest T., Smith S., Farrington K. (2004). Simulation model of renal replacement therapy: Predicting future demand in England. Nephrol. Dial. Transplant..

[3] Krishnan M., Lok C.E., Jassal S.V. (2002). Epidemiology and demographic aspects of treated end-stage renal disease in the elderly. Semin. Dial..

[4] Donovan K., Ford D., Van Schalkloyck D., Ansell D. (2009). International Comparisons with the UK RRT Programme. UK Renal Registry Report 2009.

[5] National Institute for Health & Clinical Excellence (NICE) (2008). Chronic Kidney Disease: Early Identification and Management of Chronic Kidney Disease in Adults in Primary and Secondary Care.

[6] Hagren B., Pettersen I.M., Severinsson E., Lutzen K., Clyne N. (2005). Maintenance haemodialysis: Patients’ experiences of their life situation. J. Clin. Nurs..

[7] Somma C., Matias T., Marsida K., Giorgio G. (2013). Managing end-stage renal disease in the elderly. Aging Health.

[8] Murtagh F., Spagnolo A.G., Panocchia N., Gambaro G. (2013). Conservative (non dialytic) management of end-stage renal disease and withdrawl of dialysis. Prog. Palliat. Care.

[9] Murtagh F., Addington-Hall J.M., Edmonds P. (2007). Symptoms in advanced renal disease-a cross sectional survey of symptoms prevalence in stage 5 chronic kidney disease managed without dialysis. J. Palliat. Med..

[10] Munshi S.K., Vijayakumar N., Taub N.A., Bhullar H., Lo T.C., Warwick G. (2001). Outcome of renal replacement therapy in the very elderly. Nephrol. Dial. Transplant..

[11] Burns A. (2006). So what does ‘Conservative Care’ for renal patients really mean?. Kidney Life.

[12] Department of Health (2013). Review of Liverpool Care Pathway for Dying Patients.

[13] Leadership Alliance for the Care of Dying People (2014). Priorities of Care for the Dying Person.

[14] The Royal Free Palliative Care Team Excellent Care in the Last Days of Life for Adults over 18: Practical guidance. http://freenet/freenetcms/Default.aspx?&s=38&p=1060&m=1739.

[15] Coalition for Supportive Care of Kidney Patients Best Practices for Symptom Management. http://kidneysupportivecare.org/Home.aspx.

[16] Department of Health (2005). The National Service Framework for Renal Services: Chronic Kidney Disease, Acute Renal Failure and End of Life Care.

[17] Department of Health (2008). End of Life Care Strategy—Promoting High Quality Care for All Adults at the End of Life.

[18] Harrison K., Watson S. (2011). Palliative care in advanced kidney disease: A nurse-led joint renal and specialist palliative care clinic. Int. J. Palliat. Nurs..

[19] Kane P., Vinen K., Murtagh F. (2013). Palliative care for advanced renal disease: A summary of the evidence and future direction. Palliat. Med..

